# Evaluation of offline adaptive planning techniques in image‐guided brachytherapy of cervical cancer

**DOI:** 10.1002/acm2.12462

**Published:** 2018-10-03

**Authors:** Han Liu, James Kinard, Jacqueline Maurer, Qingyang Shang, Caroline Vanderstraeten, Lane Hayes, Benjamin Sintay, David Wiant

**Affiliations:** ^1^ Department of Radiation Oncology Cone Health Cancer Center Greensboro NC USA

**Keywords:** Image‐guided brachytherapy, offline adaptive planning

## Abstract

Modern three‐dimensional image‐guided intracavitary high dose rate (HDR) brachytherapy is often used in combination with external beam radiotherapy (EBRT) to manage cervical cancer. Intrafraction motion of critical organs relative to the HDR applicator in the time between the planning CT and treatment delivery can cause marked deviations between the planned and delivered doses. This study examines offline adaptive planning techniques that may reduce intrafraction uncertainties by shortening the time between the planning CT and treatment delivery. Eight patients who received EBRT followed by HDR boosts were retrospectively reviewed. A CT scan was obtained for each insertion. Four strategies were simulated: (A) plans based on the current treatment day CT; (B) plans based on the first fraction CT; (C) plans based on the CT from the immediately preceding fraction; (D) plans based on the closest anatomically matched previous CT, using all prior plans as a library. Strategies B, C, and D allow plans to be created prior to the treatment day insertion, and then rapidly compared with the new CT. Equivalent doses in 2 Gy for combined EBRT and HDR were compared with online adaptive plans (strategy A) at *D*_90_ and *D*_98_ for the high‐risk CTV (HR‐CTV), and *D*_2 cc_ for the bladder, rectum, sigmoid, and bowel. Compared to strategy A, *D*_90_ deviations for the HR‐CTV were −0.5 ± 2.8 Gy, −0.9 ± 1.0 Gy, and −0.7 ± 1.0 Gy for Strategies B, C, and D, respectively. *D*_2 cc_ changes for rectum were 2.7 ± 5.6 Gy, 0.6 ± 1.7 Gy, and 1.1 ± 2.4 Gy for Strategies B, C, and D. With the exception of one patient using strategy B, no notable variations for bladder, sigmoid, and bowel were found. Offline adaptive planning techniques can shorten time between CT and treatment delivery from hours to minutes, with minimal loss of dosimetric accuracy, greatly reducing the chance of intrafraction motion.

## 1 INTRODUCTION

Modern three‐dimensional image‐guided intracavitary high‐dose rate (HDR) brachytherapy is increasingly used in combination with external beam radiotherapy (EBRT) and/or chemotherapy to manage cervical cancer worldwide, with significant improvement of local disease control and survival reported.^1^, ^2^, ^3^, ^4^ The entire radiation treatment is typically delivered in 45 Gy for 25 fractions with EBRT followed by HDR in 4–6 fractions using the tandem and ring (T&R) or tandem and ovoid (T&O) applicators. Magnetic resonance imaging^5^, ^6^, ^7^, ^8^ or computerized tomography (CT)^9^, ^10^, ^11^ are currently used in HDR treatment planning to define the applicator position and delineate the target and organs at risk (OARs).

There are several uncertainties in the course of HDR treatment which could result in deviations between the actual delivered and the planned doses, including source calibration, dose calculation accuracy, target and OARs delineation, inter‐fraction, and intra‐fraction motions, etc. Source calibration, dose calculation, and contour delineation uncertainties have been extensively studied in the literature, and are beyond the scope of this study.^12^ During the course of the HDR treatment, since the target, OARs and HDR applicator are not a rigid system, their relative positions may change not only from treatment to treatment, but also between the image acquisition and treatment delivery. A recent failure modes and effects analysis (FMEA) study identified the potential for applicator movement as one of the most high‐ranking failure modes in the HDR treatment.^13^ Due to the steep dose gradient in HDR treatment planning, small changes in the relative position between regions of interest and the applicator could lead to marked differences between the actual delivered and the planned doses. In this work we will focus on the inter‐ and intrafraction motion uncertainties during the T&R HDR treatment of cervical cancer.

Currently popular clinic practices for HDR treatment include using a single plan to treat the patient throughout the course, or creating an online adaptive plan for each fraction. In the single plan strategy, the plan from the first fraction can be propagated to the remaining treatments under the assumption that interfraction motion can be ignored. The intrafraction motion between the applicator insertion and treatment delivery can be minimized from the second fraction onward for the single plan strategy. However, previous studies have shown that the interfraction motion of critical structures relative to the applicator may cause marked dose deviations between the planned and delivered dose.^14^ Under the assumption that variations due to interfraction motion are much greater than those due to the intrafraction motion, online adaptive replanning on a per fraction basis have been implemented for HDR treatment.^15^, ^16^ Online adaptive planning techniques can eliminate the interfraction motion since a new CT image will be acquired for the treatment planning each day. However, the time between the image acquisition and treatment delivery can be several hours. Significant anatomic changes may occur during that time period, and could increase the uncertainty in dosimetry. Dosimetric comparisons between single planning and adaptive daily planning strategies have been investigated, and improved dose sparing for OARs has been found for the adaptive daily planning technique.^17^

However, the dosimetric impact of week‐to‐week interfraction motion versus a few hours of intrafraction motion is still under debate. Recently, the results of a large, multi‐institution study suggest that effects of inter‐ and intrafraction motion may not be as different as we once believed.^18^ The purpose of this study was to evaluate adaptive offline replanning techniques which may potentially reduce the operating room to treatment completion time for the HDR treatments, thus minimizing both inter‐ and intrafraction motion.

## 2 MATERIALS AND METHODS

Eight locally advanced cervical cancer patients (two patients with stage IB2, six patients with stage III‐B) treated between March 2016 and May 2017 were retrospectively reviewed in this study. All patient data were collected in an institutional review board‐approved registry. All patients received EBRT for a total dose of 45 Gy in 25 fractions with three‐dimensional conformal technique, then followed by five fractions of T&R HDR brachytherapy boosts with a prescription dose of 5.5 Gy per fraction. All patients in this study received a CT scan for HDR planning. For each HDR treatment, a planning CT scan with 1‐mm slice thickness was acquired for each T&R insertion, and the regions of interest, including the high‐risk clinical target volume (HR‐CTV), rectum, bladder, sigmoid, and small bowel, were manually contoured on the planning CT image. The clinical plan was created in the BrachyVision (version 15) of Eclipse treatment planning system (Varian Medical System, Palo Alto, CA), and delivered at the GammaMed HDR afterloader platform. The HDR plan quality was accessed by using the biologically equivalent doses in 2 Gy fractions (EQD_2_) of the combined EBRT and HDR plans based on the linear quadratic model:

$$EQD_{2}=\frac{\mathrm{BED}}{\left(1+\frac{2}{\alpha /\beta }\right)},$$

$$BED=nd\left(1+\frac{d}{\alpha /\beta }\right).$$

where *n* is the number of fractions, *d* is the dose per fraction, and *α/β *= 10 Gy for the HR‐CTV and 3 Gy for OARs. Our in‐house guideline for the combined EBRT and HDR treatments is to maintain the dose *D*_2 cc_ (the minimum doses to the highest irradiated 2 cc volume) <90 Gy for the bladder, and *D*_2 cc_ <75 Gy for all other OARs (rectum, sigmoid, and small bowel),^19^ while keeping the HR‐CTV coverage *D*_90_ (dose to 90% of target volume) >80 Gy.

**Figure 1:**
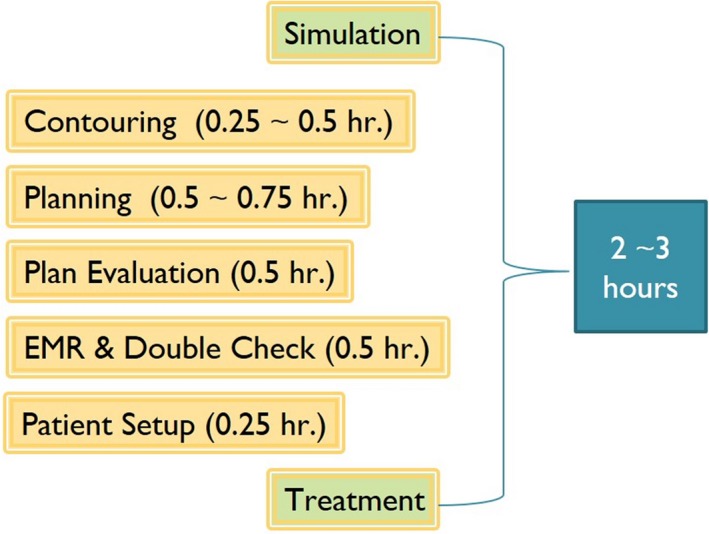
Current brachytherapy workflow/timeline.

**Figure 2:**
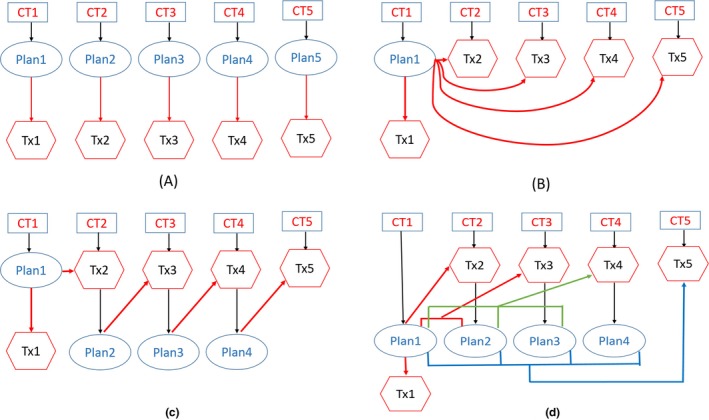
Simulation of four different workflow for the HDR treatment. (a) Online adaptive strategy; (b) Single plan strategy; (c) Offline adaptive strategy based on the immediately preceding image; (d) Offline adaptive strategy based on the closest anatomically matched prevous image.

Figure 1 shows the workflow/timeline for a T&R HDR treatment in our practice. A typical HDR T&R treatment takes about 2–3 h from the time of image acquisition to the end of treatment delivery. During this time period, noticeable anatomic changes of the regions of interest relative to the applicator (intrafraction motion) yields dosimetric uncertainty due to the sharp dose gradient in the HDR treatment plan. In order to minimize dosimetry uncertainty due to the intrafraction motion, we proposed two offline adaptive replanning strategies. The results from both offline strategies together with single plan strategy were compared with the clinical online daily adaptive replanning strategy (see Fig. 2 for an illustration).

### 2.A Strategy A

Online adaptive replanning strategy. A new CT is acquired. Target and OARs are manually contoured, and a new plan is created for each fraction.

### 2.B Strategy B

Single plan strategy. No new CT is required. The treatment plan from the first fraction is delivered for the rest of the treatment course.

### 2.C Strategy C

Offline adaptive replanning strategy. A new CT is acquired. The new CT is rigidly registered to the CT from the immediately preceding fraction. The contours and plan from preceding fraction are transferred to the current CT. Contours are edited as needed. The dose from the previous plan is evaluated on the current CT. After the treatment, a new offline plan is generated with the newly acquired CT and will be used for next treatment delivery.

### 2.D Strategy D

Offline adaptive replanning strategy. A new CT is acquired. The new CT is registered to CTs from all previous fractions. The contours and plans from the previous fractions are transferred to the current CT. Contours are edited as needed. The doses from previous plans are evaluated on the current CT, and the best plan will be picked for the treatment day delivery.

The doses to the HR‐CTV and critical organs were compared for strategies B/C/D versus strategy A. The dose metrics *D*_98_ and *D*_90_ were evaluated for the HR‐CTV, and *D*_2 cc_ was evaluated for the bladder, rectum, sigmoid, and small bowel. The dosimetric parameters (*D*_98_, *D*_90_, and *D*_2 cc_) of each HDR treatment were found by transferring the doses from prior plans after rigid registration of the prior and current CTs (registrations were based on the T&R applicator). All registrations were performed in MIM Maestro (MIM software, Cleveland, OH, USA). The paired student's *t*‐tests were applied to evaluate the significance of the comparison.

Opposed to strategy A's online adaptive planning method, strategies C and D use offline adaptive planning techniques. Both offline adaptive strategies utilize previously available plans and therefore substantially reduce the time between the T&R applicator insertion and treatment delivery, thus minimizing the intrafraction uncertainty.

For five out eight patients in this study, the same T&R applicator was used throughout the course of brachytherapy. For the other three patients, a different T&R applicator was used for the first treatment, so strategies B, C, and D applied from the second fraction.

## 3 RESULTS

**Figure 3:**
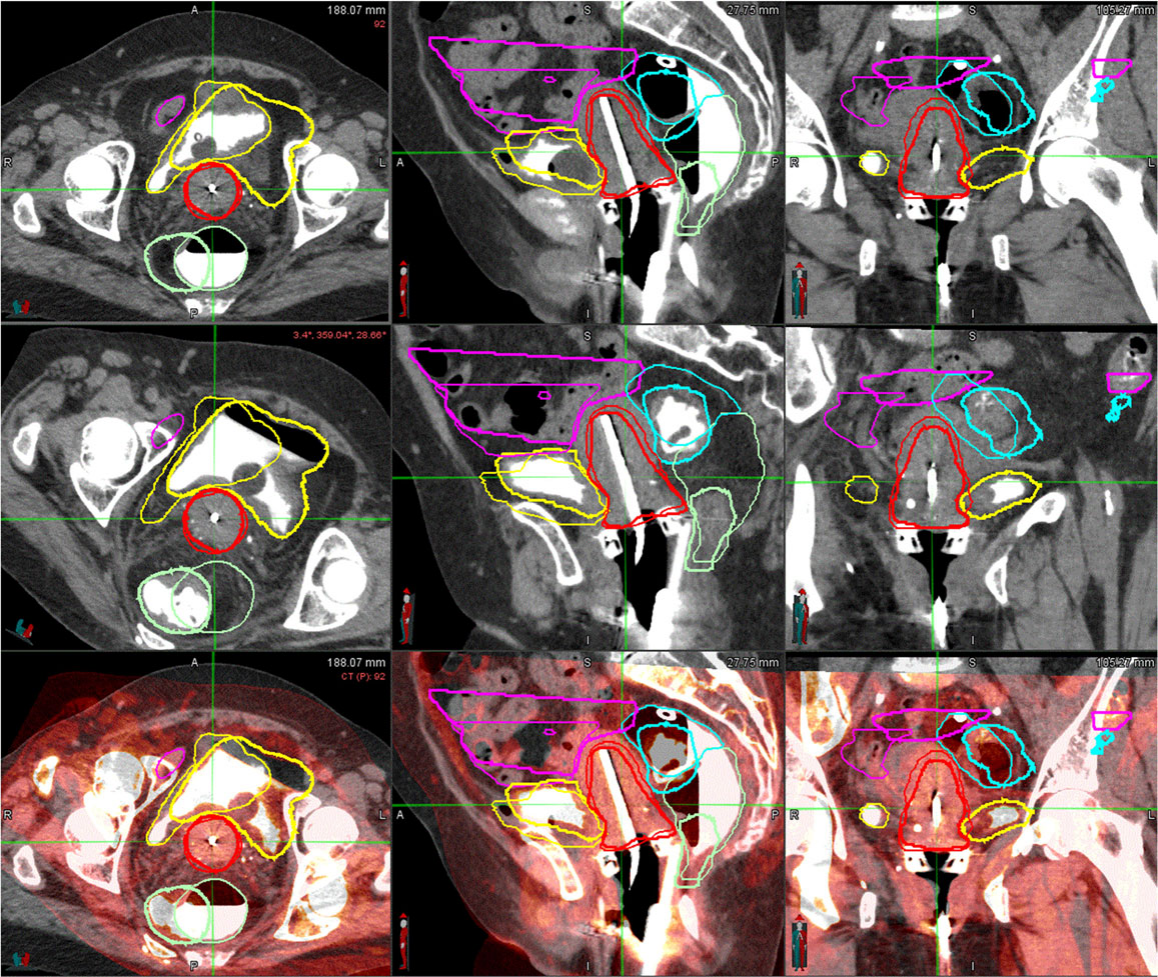
Contour comparison of HR‐CTV and organs at risk after rigid image registration based on the tandem and ring (T&R) applicator for a representative patient. Red: HR‐CTV; Yellow: Bladder; Green: Rectum; Blue: Sigmoid; Purple: Small Bowel.

Figure 3 shows the contour variation of the target and OARs for two adjacent treatments of a representative patient after the rigid registration based on the T&R applicator. Since the registration is based on the T&R applicator, only small interfraction motion was observed for the HR‐CTV. However, significant day‐to‐day variations of the OARs were found due to their filling changes.

Only one patient (Patient #7) marginally failed to meet the small bowel constraint due to the anatomy of that patient. Table 1 shows deviations of EQD_2_ in Gy for the HR‐CTV and OARs between the daily online adaptive planning (strategy A) and the other three planning strategies (B, C, and D), averaged over all eight patients. Compared to the clinically used plan (strategy A), a small variation in the HRCTV coverage was observed for all three other strategies simulated but still acceptable (within 3%). With regard to the critical organs, markedly more deviations of rectum, sigmoid, and bowel from daily online planning were observed for strategy B versus strategies C and D. The same patient that failed to meet the bowel constraint clinically also violated the bowel constraint for all three other strategies. For all other patients in this study, all constraints were met in all cases for offline adaptive strategies C and D, but there were two instances of violated constraints using strategy B (Patient #5 for bladder and Patient #2 for rectum).

**Table 1:** Deviations of the target coverage and doses to the critical structures in cumulative EQD_2_ (in Gy) between the single plan/offline adaptive strategies (strategies B, C, and D) and daily online planning strategy (strategy A)

Strategy	HR‐CTV (*D*_90_)	HR‐CTV (*D*_98_)	Bladder (*D*_2 cc_)	Rectum (*D*_2 cc_)	Sigmoid (*D*_2 cc_)	Bowel (*D*_2 cc_)
B	−0.5 ± 2.8	−0.6 ± 2.2	0.4 ± 4.0	2.7 ± 5.6	1.5 ± 3.4	1.6 ± 3.1
C	−0.9 ± 1.0	−0.7 ± 0.9	0.6 ± 2.0	0.6 ± 1.7	0.6 ± 1.4	0.3 ± 0.8
D	−0.7 ± 1.0	−0.4 ± 0.9	0.1 ± 2.0	1.1 ± 2.4	0.5 ± 1.0	0.5 ± 0.9

**Figure 4:**
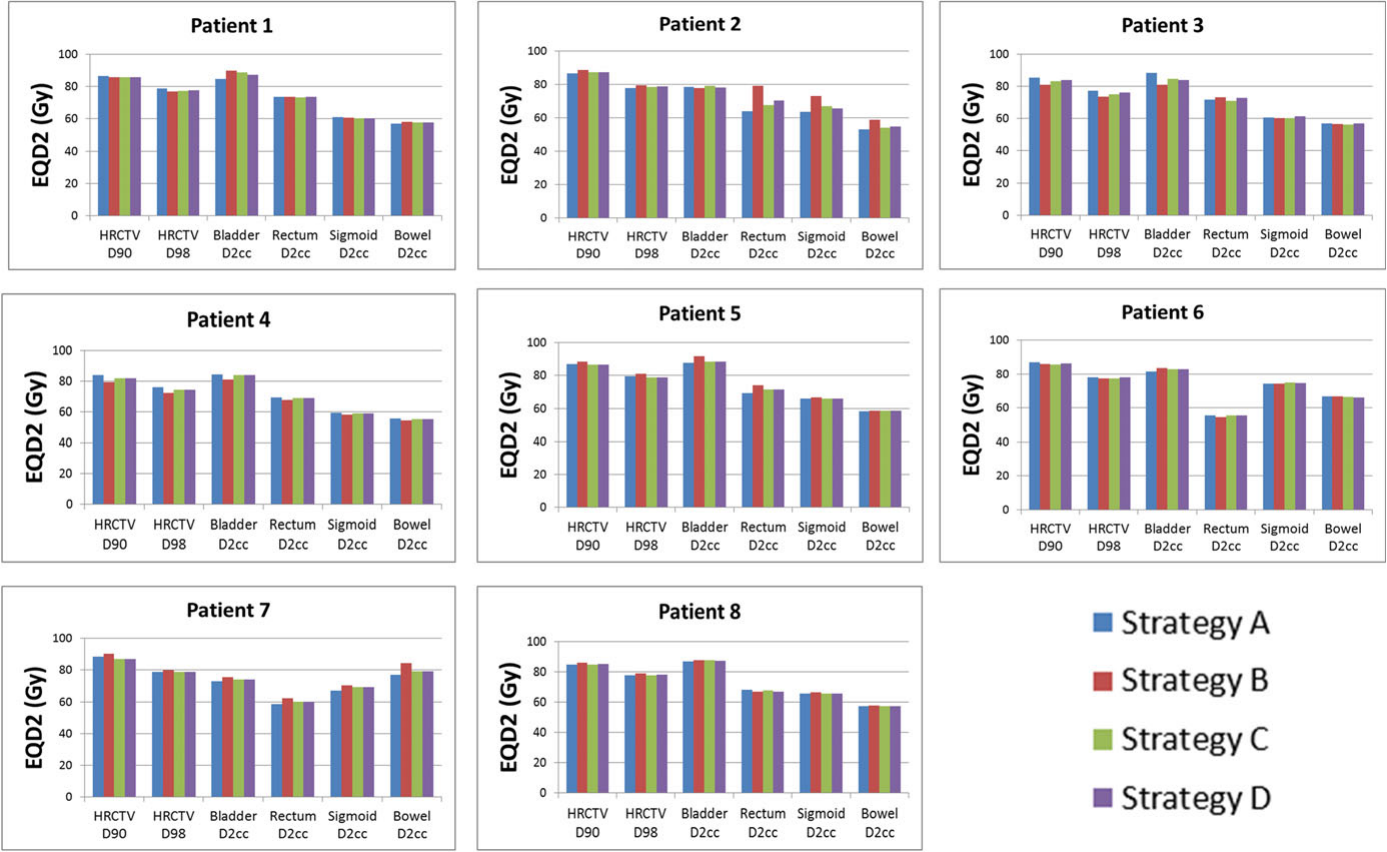
Comparison of equivalent doses in 2 Gy at the end of treatment for the HR‐CTV and OARs from four planning strategies.

Figure 4 compares cumulative EQD_2_ for the HR‐CTV and OARs for different strategies for each individual patient simulated in this study. No statistical significant difference was found between the clinical plan (strategy A) and other three strategies in terms of HR‐CTV coverages (*D*_98_ and *D*_90_). The *p*‐value from the paired student *t*‐test is >0.3 for *D*_90_ and *D*_98_ for strategies B, C, and D in all cases.

For five out of eight patients in this study, all plans from strategy C were identical to those of strategy D, indicating that the immediately preceding CT is the closest anatomically matched previous CT from the library. For the other three patients, only one plan showed deviation between strategy C and strategy D.

## 4 DISCUSSIONS

Three‐dimensional image‐guided intracavitary HDR brachytherapy is becoming an increasingly popular treatment option for cervical cancer in combination with EBRT due to its improvement of local disease control and survival. Dosimetric uncertainties in the context of HDR brachytherapy are different from that of EBRT due to the steep dose gradients in the HDR treatment. Therefore, one of the largest uncertainties in the HDR treatment is the inter‐ and intrafraction motion during the course of treatment. Applicator displacements relative to important anatomical structures can occur between different insertions or between the insertion and treatment delivery. Small changes in the relative position of the critical structures and the HDR applicator could lead to large changes in the dosimetry in the organs at risk. Two currently popular clinical practices for HDR planning involve either using a single plan technique or an online adaptive replanning technique. Each of these techniques has their advantages and disadvantages. The single plan technique ignores the interfraction motion while the online adaptive technique could have large intrafraction motion due to the hour‐long delay between the applicator insertion and treatment delivery. In this work we examined several offline adaptive planning techniques for T&R HDR treatment. These offline adaptive techniques not only reduce the patient waiting time thus minimizing the intrafraction motion, but also partially account for interfraction motion by choosing the mostly similar patient anatomy/plan. It inherits advantages from both single plan and online adaptive techniques. The dosimetry was compared between the single plan and online adaptive replanning strategies.

Compared to clinical online adaptive replanning, there was no statistically significant difference in target coverage for all other three techniques simulated in this study. The preservation of target coverage can be attributed to the fact that the target and HDR applicator remained as a rigid system throughout the HDR treatment course. With regard to the critical organs, only one patient (patient #7) failed to meet the small bowel constraint clinically. This patient also violated the bowel constraint for all other simulated strategies in the study, indicating that no adaptive planning strategy can fully offset unfavorable anatomical positions during radiation therapy. For all other patients studied, there were violations for the critical organs for strategy B (one case for bladder and one case for rectum), but no violations for any critical organs were observed for strategies C and D.

While strategy B is the only method which substantially decreases planning efforts, our data showed it had the most uncertainty. Strategies C and D do not reduce staff effort, as they require daily planning and posttreatment evaluation, but our results suggest that these strategies may provide comparable plan quality while reducing the time between applicator insertion and treatment completion, thus improving patient experience and reducing the likelihood of adverse events during the period between simulation and treatment. Using either of these two methods would, to some extent, limit the need for reduction of intrafraction motion between applicator insertion and treatment delivery. However, in cases where imaging and treatment might be done in immediate succession, the overall uncertainty involved in this process would be substantially reduced compared to the status quo.

In order to make strategies B, C and D work, the same T&R applicator should be used throughout the treatment. It should be kept in mind the proposed adaptive strategies are patient specific, for some patient with very large anatomic change between fractions, daily adaptive plan may still be necessary for the HDR treatment. The decision can be easily made after the image registration of two CT images. Alternative way to reduce the dose to the critical organ are to place vaginal packing and/or rectal paddle to increase the distance between the radioactive source and the OARs, or modify the applicator angle and ring diameter for the last treatment to reduce the doses to the OARs, as proposed by.^11^

**Figure 5:**
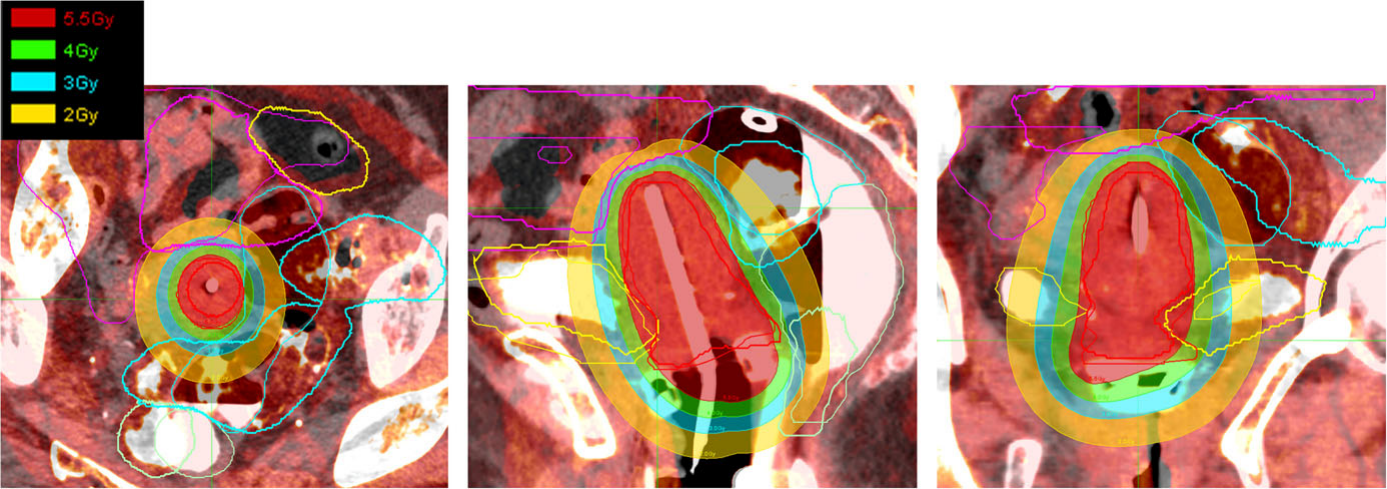
Contour comparison of regions of interest after registration based on the T&R applicator for a representative patient. Also shown the color washed isodose line in 5.5, 4, 3, and 2 Gy from the prior plan.

Even though very dramatic significant shape changes for all critical structures occurred during the treatment course (Fig. 3), we did not see large dosimetric changes at the end of the treatment for all techniques simulated in this study. This may be due to the fact that, unlike the external beam radiotherapy, only the biological equivalent doses in 2 Gy fractions of the combined HDR and EBRT to finite tissue volumes are considered until the end of the treatment course, such that the only regions near the applicator are important for the HDR dosimetric evaluation. The planner only needs to contour critical structures within certain regions, for example, 2 Gy isodose line from the prior treatment plan (as demonstrated in Fig. 5 for one representative patient). By performing this, both the intrafraction and staff effort can be reduced. Furthermore, for some patients who have significant anatomic changes on the week‐by‐week basis, with a quick image registration and partial regions of interest delineation, the DVH from the prior plan will be available for the new CT set, at that time when a decision can be made by the physician whether an online adaptive plan is necessary.

Tandem and ring (T&R) and tandem and ovoid (T&O) are the two most commonly used applicators for brachytherapy treatment of cervical cancer. Compared with T&R implantations, T&O implantations are more complex and technically harder to perform. Furthermore, there are more geometric variations for T&O than T&R due to the technical requirements of the implantation. Prior and/or continued irradiation may also lead to possible changes in morphology of the target area. As a result, the current analysis and results are only applicable in the context of T&R HDR treatments.

In current clinic practice, MRI and CT are used in HDR treatment planning to delineate the HR‐CTV and OARs. A recent analysis showed that both CT‐based and MRI‐based scans at in cervical cancer brachytherapy are adequate for OAR DVH analysis, MRI remains to be the standard for HR‐CTV definition due to its superior soft tissue contrast, and CT image can significantly overestimate the tumor volume.^20^ We want to point out in our current clinic workflow, CT images are used to define both the HR‐CTV and OARs. The evaluation of HDR treatment plan is largely driven by the OAR doses rather than the HR‐CTV coverage.

## 5 CONCLUSIONS

Offline adaptive planning techniques allow plans to be created prior to the treatment day insertion, and then rapidly compared with the new CT. Our study shows offline adaptive techniques offer similar plan quality as online adaptive strategy, while dramatically shortening the time between the CT acquisition and corresponding treatment delivery from hours to minutes, therefore improving patient experience, staff convenience, and reducing dosimetric uncertainty due to intrafraction motion.

## CONFLICT OF INTEREST

The authors declare no conflict of interest.

## References

[1] Lanciano RM , Won M , Coia LR , Hanks GE . Pretreatment and treatment factors associated with improved outcome in squamous cell carcinoma of the uterine cervix: a final report of the 1973 and 1978 patterns of care studies. Int J Radiat Oncol Biol Phys. 1991;20:667–676. 2004942 10.1016/0360-3016(91)90007-q

[2] Montana GS , Hanlon AL , Brickner TJ , et al. Carcinoma of the cervix: patterns of care studies: review of 1978, 1983, and 1988–1989 surveys. Int J Radiat Oncol Biol Phys. 1995;32:1481–1486. 7635793 10.1016/0360-3016(95)00177-Z

[3] Katz A , Eifel PJ . Quantification of intracavitary brachytherapy parameters and correlation with outcome in patients with carcinoma of the cervix. Int J Radiat Oncol Biol Phys. 2000;48:1417–1425. 11121642 10.1016/s0360-3016(00)01364-x

[4] Holloway CL , Racine ML , Cormack RA , O'Farrell DA , Viswanathan AN . Sigmoid dose using 3D imaging in cervical‐cancer brachytherapy. Radiother Oncol. 2009;93:307–310. 19665244 10.1016/j.radonc.2009.06.032 PMC2867463

[5] Potter R , Georg P , Dimopoulos JC , et al. Clinical outcome of protocol based image (MRI) guided adaptive brachytherapy combined with 3D conformal radiotherapy with or without chemotherapy in patients with locally advanced cervical cancer. Radiother Oncol. 2011;100:116–123. 21821305 10.1016/j.radonc.2011.07.012 PMC3165100

[6] Lindegaard JC , Tanderup K , Nielsen SK , Haack S , Gelineck J . MRI‐guided 3D optimization significantly improves DVH parameters of pulsed‐dose‐rate brachytherapy in locally advanced cervical cancer. Int J Radiat Oncol Biol Phys. 2008;71:756–764. 18191335 10.1016/j.ijrobp.2007.10.032

[7] Georg P , Kirisits C , Goldner G , et al. Correlation of dose‐volume parameters, endoscopic and clinical rectal side effects in cervix cancer patients treated with definitive radiotherapy including MRI‐based brachytherapy. Radiother Oncol. 2009;91:173–180. 19243846 10.1016/j.radonc.2009.01.006

[8] Kirisits C , Potter R , Lang S , Dimopoulos J , Wachter‐Gerstner N , Georg D . Dose and volume parameters for MRI‐based treatment planning in intracavitary brachytherapy for cervical cancer. Int J Radiat Oncol Biol Phys. 2005;62:901–911. 15936576 10.1016/j.ijrobp.2005.02.040

[9] Kim RY , Shen S , Duan J . Image‐based three‐dimensional treatment planning of intracavitary brachytherapy for cancer of the cervix: dose‐volume histograms of the bladder, rectum, sigmoid colon, and small bowel. Brachytherapy. 2007;6:187–194. 17606413 10.1016/j.brachy.2006.11.005

[10] Koom WS , Sohn DK , Kim JY , et al. Computed tomography‐based high‐dose‐rate intracavitary brachytherapy for uterine cervical cancer: preliminary demonstration of correlation between dose‐volume parameters and rectal mucosal changes observed by flexible sigmoidoscopy. Int J Radiat Oncol Biol Phys. 2007;68:1446–1454. 17482766 10.1016/j.ijrobp.2007.02.009

[11] Dumane VA , Yuan Y , Sheu RD , Gupta V . Computed tomography‐based treatment planning for high‐dose‐rate brachytherapy using the tandem and ring applicator: influence of applicator choice on organ dose and inter‐fraction adaptive planning. J Contemp Brachytherapy. 2017;9:279–286. 28725253 10.5114/jcb.2017.68519 PMC5509987

[12] Tanderup K , Nesvacil N , Potter R , Kirisits C . Uncertainties in image guided adaptive cervix cancer brachytherapy: impact on planning and prescription. Radiother Oncol. 2013;107:1–5. 23541642 10.1016/j.radonc.2013.02.014

[13] Mayadev J , Dieterich S , Harse R , et al. A failure modes and effects analysis study for gynecologic high‐dose‐rate brachytherapy. Brachytherapy. 2015;14:866–875. 26204807 10.1016/j.brachy.2015.06.007

[14] Kirisits C , Lang S , Dimopoulos J , Oechs K , Georg D , Potter R . Uncertainties when using only one MRI‐based treatment plan for subsequent high‐dose‐rate tandem and ring applications in brachytherapy of cervix cancer. Radiother Oncol. 2006;81:269–275. 17126938 10.1016/j.radonc.2006.10.016

[15] Beriwal S , Kim H , Coon D , et al. Single magnetic resonance imaging vs magnetic resonance imaging/computed tomography planning in cervical cancer brachytherapy. Clin Oncol (R Coll Radiol). 2009;21:483–487. 19423307 10.1016/j.clon.2009.03.007

[16] Nesvacil N , Potter R , Sturdza A , Hegazy N , Federico M , Kirisits C . Adaptive image guided brachytherapy for cervical cancer: a combined MRI‐/CT‐planning technique with MRI only at first fraction. Radiother Oncol. 2013;107:75–81. 23068712 10.1016/j.radonc.2012.09.005 PMC3675685

[17] Meerschaert R , Nalichowski A , Burmeister J , et al. A comprehensive evaluation of adaptive daily planning for cervical cancer HDR brachytherapy. J Appl Clin Med Phys. 2016;17:323–333. 27929505 10.1120/jacmp.v17i6.6408 PMC5690507

[18] Nesvacil N , Tanderup K , Hellebust TP , et al. A multicentre comparison of the dosimetric impact of inter‐ and intra‐fractional anatomical variations in fractionated cervix cancer brachytherapy. Radiother Oncol. 2013;107:20–25. 23602372 10.1016/j.radonc.2013.01.012 PMC3675683

[19] Georg P , Lang S , Dimopoulos JC , et al. Dose‐volume histogram parameters and late side effects in magnetic resonance image‐guided adaptive cervical cancer brachytherapy. Int J Radiat Oncol Biol Phys. 2011;79:356–362. 20385450 10.1016/j.ijrobp.2009.11.002

[20] Viswanathan AN , Dimopoulos J , Kirisits C , Berger D , Potter R . Computed tomography versus magnetic resonance imaging‐based contouring in cervical cancer brachytherapy: results of a prospective trial and preliminary guidelines for standardized contours. Int J Radiat Oncol Biol Phys. 2007;68:491–498. 17331668 10.1016/j.ijrobp.2006.12.021

